# Specific Strains of *Faecalibacterium prausnitzii* Ameliorate Nonalcoholic Fatty Liver Disease in Mice in Association with Gut Microbiota Regulation

**DOI:** 10.3390/nu14142945

**Published:** 2022-07-19

**Authors:** Wenbing Hu, Wenyu Gao, Zongmin Liu, Zhifeng Fang, Hongchao Wang, Jianxin Zhao, Hao Zhang, Wenwei Lu, Wei Chen

**Affiliations:** 1 State Key Laboratory of Food Science and Technology, Jiangnan University, Wuxi 214122, China; hu_wenbing@163.com (W.H.); 6200113023@stu.jiangnan.edu.cn (W.G.); 7180112023@stu.jiangnan.edu.cn (Z.L.); zhifengf@foxmail.com (Z.F.); hcwang@jiangnan.edu.cn (H.W.); jxzhao@jiangnan.edu.cn (J.Z.); zhanghao61@jiangnan.edu.cn (H.Z.); chenwei66@jiangnan.edu.cn (W.C.); 2 School of Food Science and Technology, Jiangnan University, Wuxi 214122, China; 3 National Engineering Research Center for Functional Food, Jiangnan University, Wuxi 214122, China; 4 (Yangzhou) Institute of Food Biotechnology, Jiangnan University, Yangzhou 225004, China; 5 Wuxi Translational Medicine Research Center and Jiangsu Translational Medicine Research Institute Wuxi Branch, Wuxi 214122, China

**Keywords:** nonalcoholic fatty liver disease, high-fat diet, *Faecalibacterium prausnitzii*, short-chain fatty acids, gut microbiota

## Abstract

Evidence linking *Faecalibacterium prausnitzii* abundance to nonalcoholic fatty liver disease (NAFLD) is accumulating; however, the causal relationship remains obscure. In this study, 12 *F. prausnitzii* strains were orally administered to high fat diet fed C57BL/6J mice for 12 weeks to evaluate the protective effects of *F. prausnitzii* on NAFLD. We found that five *F. prausnitzii* strains, A2-165, LB8, ZF21, PL45, and LC49, significantly restored serum lipid profiles and ameliorated glucose intolerance, adipose tissue dysfunction, hepatic steatosis, inflammation, and oxidative stress in a mouse model of NAFLD. Moreover, two strains, LC49 and LB8, significantly enhanced short-chain fatty acid (SCFA) production and modulated the gut microbiota. Based on the combined analysis of linear discriminant analysis effect size and microbial communities, the core microbiome related to NAFLD comprised *Odoribacter*, *Roseburia*, *Erysipelatoclostridium*, *Tyzzerella*, *Faecalibaculum*, *Blautia*, and *Acetatifactor*, and the last five genera can be reversed by treatment with the LC49 and LB8 strains. Additionally, the LC49 and LB8 strains enriched *Lactobacillus*, *Ileibacterium*, *Faecalibacterium*, *Dubosiella*, and *Bifidobacterium* and downregulated pathways involving carbohydrate metabolism, amino acid metabolism, and fatty acid biosynthesis. Interestingly, LC49 supplementation also upregulated tryptophan metabolism, glutathione metabolism, and valine, leucine, and isoleucine degradation, which might be related to NAFLD prevention. Collectively, *F. prausnitzii* LC49 and LB8 exerted considerable anti-NAFLD and microbiota-regulating effects, indicating their potential as probiotic agents for NAFLD treatment.

## 1. Introduction

NAFLD, the most common liver disorder characterized by fat accumulation in the liver without alcohol consumption, affects approximately a quarter of the world’s population [1]. NAFLD is considered a growing indicator for liver transplantation by 2030, substantially increasing the public health burden worldwide [2]. NAFLD is asymptomatic in most affected patients and is closely linked with an increased risk of hepatocellular carcinoma (HCC), type II diabetes, and cardiovascular disease [3,4]. To date, there are no approved drugs for NAFLD treatment, and the main clinical recommendation is weight loss with dietary changes and exercise [5].

The gut microbiota has become a critical regulator of host health in terms of energy and substrate metabolism [6]. The liver receives approximately 70–75% portal vein blood drained from mesenteric veins of the intestinal tract, making it the principal organ exposed to microorganisms and metabolites derived from the gut [7]. In 1978, the term “gut-liver axis” was initially introduced by Volta et al., whose research revealed the secretion of immunoglobulin A (IgA) antibodies to dietary antigens in individuals with liver cirrhosis, showing the strong connection between the gut and the liver [8]. A disruption of energy and substrate metabolism in the liver might be caused by abnormalities in gut bacteria composition and/or functioning [9]. For instance, trimethylamine (TMA), a byproduct of the gut microbiota’s choline metabolism, travels through the portal vein to the liver, where it can be oxidized into TMA *N*-oxide (TMAO), which might aggravate the development of NAFLD [10]. Additionally, bile acid metabolism by intestinal flora activates the nuclear bile acid receptor farnesoid X receptor (FXR) signaling, thereby affecting the progression of NAFLD [11]. Thus, the maintenance of gut microbial homeostasis is critical for the prevention of NAFLD.

To date, natural probiotic supplementation has an important impact on the host health through restoring the dysbiosis of gut microbiota, producing active substances, or shaping the gut immune responses [12,13,14]. Accumulating evidence has revealed a beneficial effect of probiotic supplementation on lipid-lowering in the liver [15,16,17]. *F. prausnitzii* is well-accepted as one of the “next-generation probiotics” (NGP), dominating in the abundance (approximately 5–15%) of total human gut bacteria [18]. Due its predominant abundance and health-beneficial characteristics, *F. prausnitzii* has been recommended as a biomarker of intestinal health [19]. Low *F. prausnitzii* abundance is closely associated with inflammatory bowel diseases (IBD), whereas supplementation with *F. prausnitzii* can reverse this disease [20,21]. Similarly, *F. prausnitzii* abundance is lower in the guts of individuals with NAFLD compared to those of healthy cohorts [22]. Accordingly, we proposed a hypothesis on a potential causative link between *F. prausnitzii* and NAFLD.

To examine this hypothesis, one reference strain, A2-165, and 11 *F. prausnitzii* isolates were orally administered to high-fat diet (HFD)-fed mice for 12 consecutive weeks. Alterations in serum lipid indicators, adipose tissue expansion, glucose intolerance, liver function enzymes, hepatic steatosis, inflammation, and oxidative stress were evaluated. Furthermore, SCFA production, gut microbial composition, and microbial functional pathways in *F. prausnitzii*-treated and untreated HFD-fed mice were compared to explore mechanistic insights into the anti-NAFLD effect of *F. prausnitzii*.

## 2. Materials and Methods

### 2.1. F. prausnitzii Strains and Culture Condition

Except for *F. prausnitzii* A2-165 (obtained from DSMZ), all strains were isolated from healthy human feces and preserved at the Jiangnan University Culture Collection of Food Microorganisms. All the participants signed an informed consent form. Detailed information on the *F. prausnitzii* strains is listed in Table S1. The bacterial suspensions used for oral administration were obtained as previously described [23].

### 2.2. Animal Experiments

Seven-week-old male C57BL/6J mice (20–22 g, SPF grade) were provided by Beijing Vital River Laboratory Animal Technology (Beijing, China). The mice were kept at a controlled temperature (23 ± 2 °C) and humidity (55 ± 10%) under a regular 12 h light–dark cycle. All experimental procedures were approved by the Animal Care and Use Committee of the Jiangnan University (JN. No. 2020930c1120130[234]).

**Figure 1:**
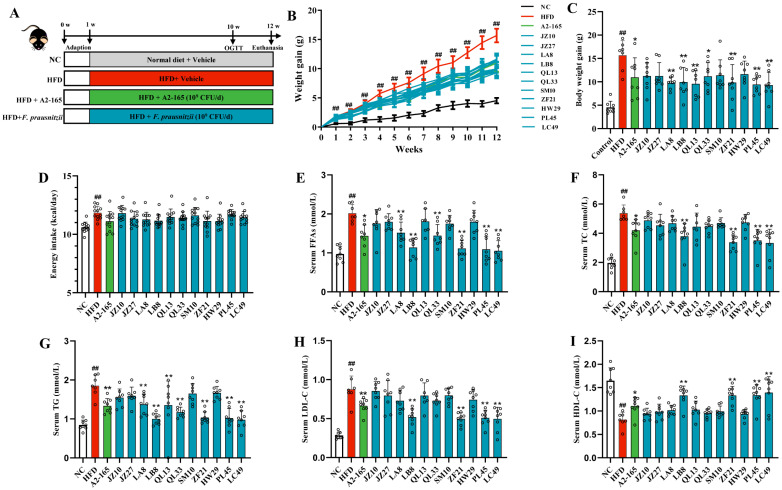
The effect of *F. prausnitzii* strains on body weight gain, energy uptake, and serum lipid profiles in NAFLD mice. (**A**) Animal groups and experimental schedule; (**B**) Changes in the body weight gain of HFD-fed mice with or without *F. prausnitzii* treatment; (**C**) Final body weight; (**D**) Energy intake in different groups; (**E**–**I**) Serum levels of FFAs, TC, TG, LDL-C, and HDL-C in different groups. Data are shown as means with SD; ^##^ *p* < 0.01 vs. NC group, unpaired Student’s *t*-test; * *p* < 0.05, ** *p* < 0.01 vs. HFD group, one-way ANOVA followed by Dunnett’s test.

After 1 week of acclimation, the mice were weighed and randomly assigned to the normal control (NC), HFD, and *F. prausnitzii*-treated groups (A2-165, JZ10, JZ27, LA8, LB8, QL13, QL33, SM10, ZF21, HW29, PL45, and LC49) (*n* = 7–8). Except for the NC group, all mice were fed an HFD for 12 weeks. The HFD (energy: 5.0 kcal/g; 20 kcal% carbohydrates, 20 kcal% protein, 60 kcal% fat) and normal control diet (NCD, energy: 3.6 kcal/g; 67.4 kcal% carbohydrates, 20.6 kcal% protein, 12 kcal% fat) were provided by Trophic (Jiangsu, China). During the treatment period, approximately 0.25 mL of bacterial suspensions (~4 × 10^9^ CFU/mL, *F. prausnitzii*-treated groups) or PBS (supplemented with 0.05% L-cysteine, NC and HFD groups) were intragastrically inoculated once a day. The preparation of bacterial suspensions for the *F. prausnitzii* strains is described in the Supplementary Materials. Body weight was recorded weekly, and food intake was documented every two days. The experimental schedule is shown in Figure 1A.

### 2.3. Measurement of Biochemical Indicators

Serum concentrations of alanine aminotransferase (ALT), aspartate aminotransferase (AST), free fatty acids (FFAs), triglycerides (TG), total cholesterol (TC), high-density lipoprotein cholesterol (HDL-C), and low-density lipoprotein cholesterol (LDL-C) were determined with a Beckman AU5800 automatic biochemical analyzer (Brea, CA, USA).

The levels of tumor necrosis factor (TNF)-α, interleukin (IL)-1β, and IL-6 in the liver were measured using ELISA kits (R&D Systems, Minneapolis, MN, USA). Glutathione peroxidase (GSH-PX), superoxide dismutase (SOD), and malondialdehyde (MDA) levels in the liver were quantified using commercial kits (Nanjing Jiancheng Bioengineering Institute, Nanjing, China).

### 2.4. Oral Glucose Tolerance Test (OGTT)

Before the OGTT, the mice fasted overnight. Blood samples collected from the tail vein’s tip were tested for glucose levels at baseline (t = 0), just prior to oral gavage of 20% (*w*/*v*) glucose (2 g/kg body weight), and then after 30, 60, 90, and 120 min. Blood glucose levels were measured using a glucometer (Accu Check; Roche, Basel, Switzerland). The insulin content was determined using an ELISA kit (CUSABIO, Wuhan, China).

### 2.5. Histological Examination

Paraffin-embedded liver and adipose tissue sections were stained with H&E or Sirius Red, and OCT-embedded frozen liver tissues were stained with Oil red O. Stained sections were scanned using a digital scanner (Pannoramic MIDI II, 3DHISTECH, Budapest, Hungary) and analyzed using an image analysis program (Image Pro-Puls, Silver Spring, MD, USA). The pathological grade of NAFLD was classified as hepatic steatosis (0, <5%; 1, 5–33%; 2, 34–66%; and 3, >66% of intracytoplasmic lipid droplets) and lobular inflammation (0, none; 1, 1–2 foci; 2, 2–4 foci; and 3, >4 foci on a ×20 microscope). All the biopsy specimens were evaluated by two independent experts.

### 2.6. SCFA Metabolism

Feces were freeze-dried and weighed, followed by SCFA analysis according to Mao et al. [24].

### 2.7. Metagenomic Analysis

Total DNA from stool samples was obtained using a Fast DNA Stool Kit (MP Biomedicals, CA, USA) and applied to amplify the 16s rDNA V3-V4 region. The amplicons were purified and sequenced using the Illumina Miseq platform (Illumina, Santiago, CA, USA). The detailed method of gut microbiota analysis is described in the Supplementary Materials.

### 2.8. Statistical Analysis

SPSS 22.0 (IBM Corporation, Somers, NY, USA) was applied to perform all the statistical analyses. All values are expressed as means ± SD. Unpaired Student’s *t*-tests were performed between the NC and HFD groups, and a one-way ANOVA was performed to compare the effects of *F. prausnitzii* in HFD-treated animals, followed by Dunnett’s multiple comparison test against the HFD group. *p* < 0.05 was considered statistically significant. 

## 3. Results

### 3.1. F. prausnitzii Supplementation Decreased HFD-Induced Weight Gain and Hyperlipidemia in HFD-Fed Mice

To examine the causality between NAFLD and *F. prausnitzii*, 12 *F. prausnitzii* strains (including the reference strain A2-165 and 11 *F. prausnitzii* isolates) were orally administered to HFD-fed mice for 12 weeks. At the first week, the body weight gain of mice in the HFD group began to be remarkedly higher than that of the mice in the NC group (Figure 1B; *p* < 0.01). However, *F. prausnitzii* treatment, except for the JZ10, JZ27, SM10, and HW29 strains, significantly reduced the body weight gain at 12 weeks of feeding compared to that of the HFD-fed mice without treatment (Figure 1C; *p* < 0.05–0.01). No statistically significant differences were observed in the energy intake per mouse between the HFD and *F. prausnitzii*-treated groups (Figure 1D; *p* > 0.05). Moreover, supplementation with *F. prausnitzii* strains, including A2-165, LB8, ZF21, PL45, and LC49, remarkably reduced serum FFAs, TC, TG, and LDL-C concentrations and elevated HDL-C content compared to the mice in the HFD group (Figure 1E–I; *p* < 0.01–0.05).

### 3.2. F. prausnitzii Supplementation Ameliorated Adipose Tissue Dysfunction and Glucose Intolerance in HFD-Fed Mice

**Figure 2:**
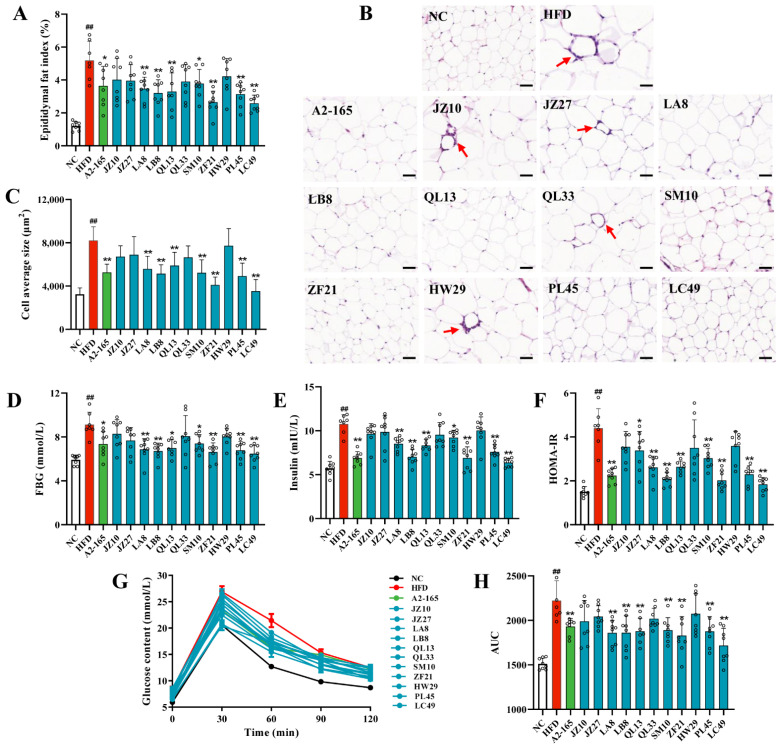
*F. prausnitzii* treatment reduced fat accumulation and improved insulin resistance in NAFLD mice. (**A**) Epididymal fat index; (**B**) Representative H&E staining of epididymal fat tissue (200×, scale bars represent 50 μm, red arrow indicates inflammation); (**C**) Measurement of adipocyte cell sizes; (**D**) Fasting blood glucose (FBG) level; (**E**) Fasting insulin (FINS) level; (**F**) HOMA-IR index, FBG * FINS/22.5; (**G**) Means of blood glucose levels at 0, 30, 60, 90, and 120 min after oral gavage of 20 % (*w*/*v*) glucose (2 g/kg body weight); (**H**) Means of area under the concentration curve (AUC). Data are shown as means with SD; ^##^ *p* < 0.01 vs. NC group, unpaired Student’s *t*-test; * *p* < 0.05, ** *p* < 0.01 vs. HFD group, one-way ANOVA followed by Dunnett’s test.

Adipocyte hypertrophy and inflammation are hallmarks of adipose tissue dysfunction. Therefore, the epididymal fat index, adipocyte size, and inflammatory cytokine-related gene expression were determined in this study. As shown in Figure 2A, the epididymal fat index in the HFD group was notably increased compared to the NC group (*p* < 0.01). However, the *F. prausnitzii*-treated groups, except for the JZ10, JZ27, QL33, and HW29 groups, showed a remarkably lower fat index than the HFD group (*p* < 0.01–0.05). Moreover, the HFD-induced adipocyte expansion and inflammation were also reversed following treatment with the *F. prausnitzii* strains, except for JZ10, JZ27, QL33, and HW29 (Figure 2B, C; *p* < 0.01). Similarly, mice treated with *F. prausnitzi* showed lower mRNA expression of TNF-α and IL-6 than those of the HFD group, which was in accordance with the results of the adipose tissue dysfunction (Table S2; Figure S2).

Furthermore, we evaluated the effects of *F. prausnitzii* treatment on glucose intolerance in HFD-fed mice. Long-term HFD-fed mice showed significantly higher fasting blood glucose (FBG), fasting insulin, and HOMA-IR values than NCD-fed mice (Figure 2D–F; *p* < 0.01). However, the values of these three indicators were reversed by the *F. prausnitzii* treatment (*p* < 0.01–0.05), except for the JZ10, JZ27, QL33, and HW29 strains. Likewise, impaired glucose tolerance was also restored in mice treated with these *F. prausnitzii* strains (Figure 2G,H; *p* < 0.01) compared with the mice in the HFD group.

### 3.3. F. prausnitzii Supplementation Prevented Liver Injury and Hepatic Steatosis in HFD-Fed Mice

**Figure 3:**
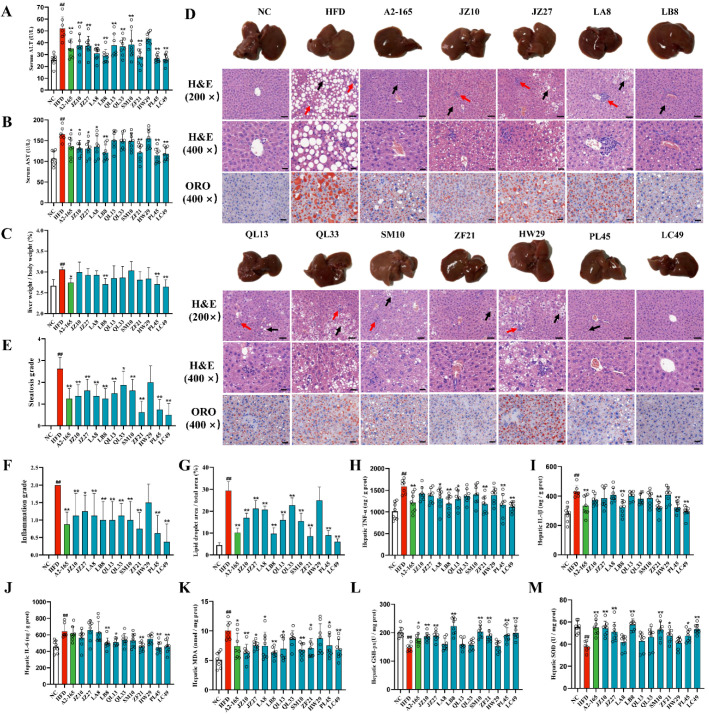
*F. prausnitzii* treatment prevented hepatic steatohepatitis and liver injury in NAFLD mice. (**A**) Serum ALT content; (**B**) Serum AST content; (**C**) Liver index; (**D**) Representative H&E and Oil red O stains of liver sections (scale bars represent 20 and 50 μm for 400× and 200×, respectively), black arrow indicates lipid droplet, steatosis, and cytoplasmic vacuoles, and red arrow indicates lobular inflammation; (**E**) Steatosis grade; (**F**) Inflammation grade; (**G**) Quantification of Oil red O stained liver lipid droplets in (**D**); (**H**–**K**) Hepatic TNF-α, IL-1β, IL-6, and MDA levels in different groups; (**L**,**M**) Hepatic GSH-PX and SOD activities in different groups. Data are shown as means with SD; ^##^ *p* < 0.01 vs. NC group, unpaired Student’s *t*-test; * *p* < 0.05, ** *p* < 0.01 vs. HFD group, one-way ANOVA followed by Dunnett’s test.

Excess dietary fat promotes excessive fat deposition in the liver, which causes hepatic steatosis, leading to oxidative stress and inflammatory reactions in the hepatocytes [25]. The serum AST and ALT levels are commonly considered sensitive indicators of liver injury. The HFD remarkedly elevated the serum ALT and AST levels in the mice, whereas treatment with *F. prausnitzii* effectively reversed the levels of these two indicators (Figure 3A,B; *p* < 0.01–0.05), except for the QL13, QL33, SM10, and HW29 strains. Moreover, the liver indices in the A2-165, LB8, PL45, and LC49 groups were markedly decreased compared with the HFD group (Figure 3C; *p* < 0.01–0.05). Regarding the liver pathology of the HFD-fed mice, the structure of hepatic lobules was distorted, and the area of intracytoplasmic lipid droplets accounted for >50% of the field of view, accompanied by inflammatory cell infiltration in the hepatic lobules (Figure 3D–F). Strikingly, *F. prausnitzii* treatment, except for the HW29 strain, significantly improved the hepatic pathology, which was confirmed by the quantification of the fibrotic area and lipid area per total area (Figure 3G and Figure S3; *p* < 0.01).

To evaluate the hepatic oxidative stress and inflammatory reactions, the SOD and GSH-PX activities and MDA, TNF-α, IL-1β, and IL-6 levels were determined. As shown in Figure 3H–M, the HFD group presented a high activation of hepatic oxidative stress and inflammatory processes, which was reflected by the significantly increased levels of TNF-α, IL-1β, IL-6, and MDA as well as decreased SOD and GSH-PX activities. Among the 12 *F. prausnitzii* strains, only four strains, including LB8, ZF21, PL45, and LC49, restored all six indicators. 

### 3.4. F. prausnitzii Supplementation Regulated the Gut Microbial Composition in HFD-Fed Mice

**Figure 4:**
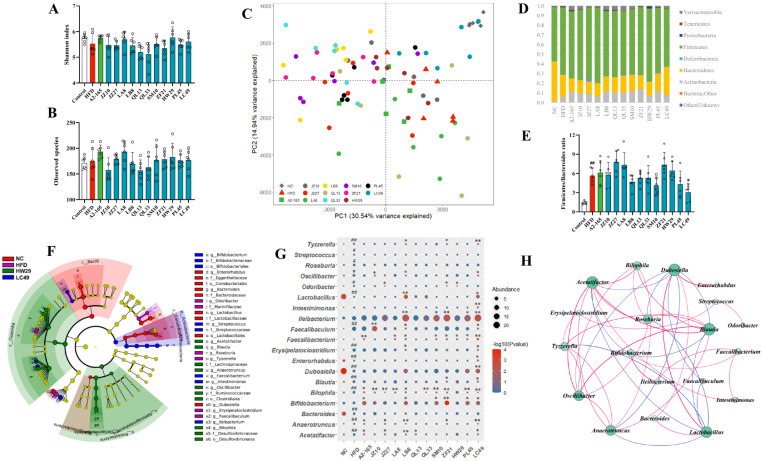
The effect of *F. prausnitzii* strains on gut microbial composition in NAFLD mice. (**A**,**B**) Shannon index and observed species; (**C**) Principal coordinates analysis; (**D**) Changes in the abundance of phylum level; (**E**) The ratio of Firmicute/Bacteroidetes; (**F**) Cladogram depicting the differentially abundant taxa (LDA score > 2 and *p* < 0.05) among all groups. The right panel presents the differential taxa labeled with various tags; (**G**) The relative abundance of taxa remarkedly changed by HFD feeding and *F. prausnitzii* treatment. The size and color of the circles display the average abundance and *p* value, respectively; (**H**) Interaction network analysis among microbial biomarkers. The circle size indicates the contribution, and the line color represents a positive (red) or negative correlation (blue), respectively (*p* < 0.05, r > 0.3 or r < −0.3). Data are shown as means with SD; ^#^ *p* < 0.05, ^##^ *p* < 0.01 vs. NC group, unpaired Student’s *t*-test; * *p* < 0.05, ** *p* < 0.01 vs. HFD group, one-way ANOVA followed by Dunnett’s test.

Furthermore, we determined the effect of *F. prausnitzii* strains on the gut microbial composition based on the 16s-RNA-amplicon sequencing. *F. prausnitzii* treatment did not change the α-diversity among the NC, HFD, and *F. prausnitzii*-treated groups, with no significant differences observed in the Shannon index and observed species (Figure 4A,B). However, the distinction in β-diversity between the NC and HFD groups was evident in the PCA analysis (Figure 4C). Among the 12 *F. prausnitzii* strains, only the LC49 group was close to the NC group, indicating the restorative effect of LC49 on HFD-induced gut microbial dysbiosis. The dominant phyla in all groups were *Firmicutes*, *Bacteroides*, and *Actinobacteria* (Figure 4D). The HFD-fed group showed a significantly higher ratio of *Firmicutes*/*Bacteroides* compared to the NCD-fed mice (Figure 4E; *p* < 0.01), whereas LC49 treatment normalized this ratio (*p* < 0.05).

Gut microbial biomarkers were explored using LEfSe. As shown in Figure 4F, 19 genera were identified as microbial biomarkers among the NC, HFD, HW29, and LC49 groups. The core microbiome in the HFD group comprised *Tyzzerella*, *Roseburia*, *Odoribacter*, *Faecalibaculum*, and *Erysipelatoclostridium* (Figure 4F). The comparison analysis revealed that the HFD group showed a significantly higher abundance of *Tyzzerella*, *Streptococcus*, *Roseburia*, *Oscillibacter*, *Odoribacter*, *Faecalibaculum*, *Erysipelatoclostridium*, *Blautia*, *Bilophila*, *Anaerotruncus*, and *Acetatifactor* and a lower abundance of *Dubosiella*, *Lactobacillus*, *Enterorhabdus*, and *Bacteroides* than the NC group (Figure 4G; *p* < 0.05–0.01). Of the 12 *F. prausnitzii* strains, LB8 and LC49 not only enriched *Bifidobacterium*, *Lactobacillus*, *Dubosiella*, *Faecalibacterium*, and *Ileibacterium* but also restored 8 and 11 HFD-dependent taxa, respectively (Figure 4G). However, the quantification analysis suggested that *F. prausnitzii* isolates could be varied in their ability to colonize the mouse gut (Figure S4). The potential interactions among these biomarker genera were investigated using a network analysis. As shown in Figure 4H, *Acetatifactor*, *Blautia*, *Lactobacillus*, and *Dubosiella* were the potential hub genera among the 19 biomarker genera. Moreover, *Faecalibacterium* showed a strong positive relationship with *Bifidobacterium*, *Ileibacterium*, and *Intestinimonas* (Figure 4H; r > 0.3, *p* < 0.05).

### 3.5. F. prausnitzii Supplementation Changed Metagenomic Functions and SCFA Production in HFD-Fed Mice

**Figure 5:**
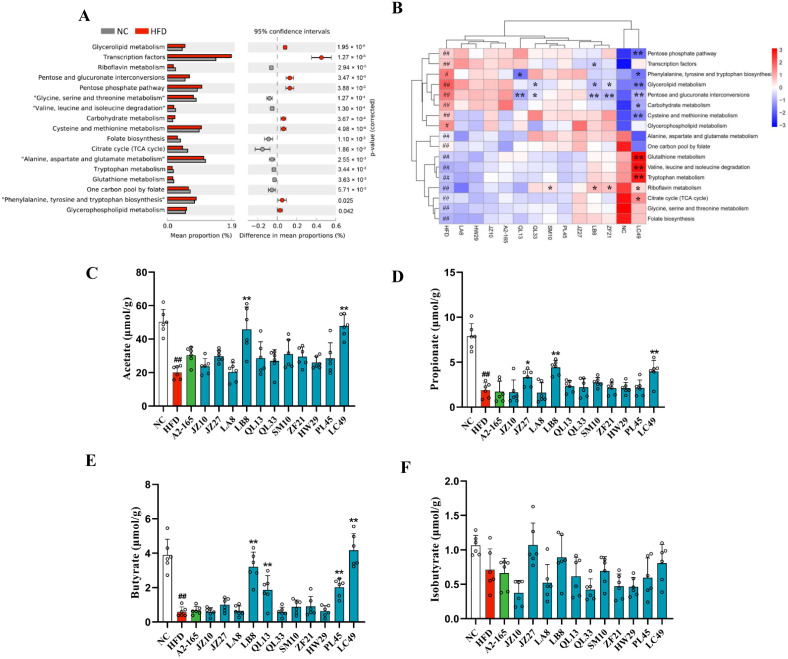
Prediction of microbial functional pathways and short-chain fatty acid production. (**A**) Differential pathways between the NC and HFD groups (two-sided Welch’s *t*-test at *p* < 0.05); (**B**) Heatmap of the differential pathways in (**A**) among all groups; (**C**–**F**) Colonic acetate, propionate, butyrate, and iso-butyrate concentrations. Data are shown as means ± SD; ^#^ *p* < 0.05, ^##^ *p* < 0.01 vs. NC group, unpaired Student’s *t*-test; * *p* < 0.05, ** *p* < 0.01 vs. HFD group, one-way ANOVA followed by Dunnett’s test.

To investigate the impact of *F. prausnitzii* supplementation on the metagenomic functions of gut microbiota, a PICRUSt analysis was performed. Based on Welch’s test, 17 significantly different pathways were observed between the NC and HFD groups (Figure 5A; *p* < 0.01–0.05). The comparisons among all groups showed that pathways involving riboflavin metabolism; glutathione metabolism; valine, leucine, and isoleucine (BCAAs) degradation; tryptophan metabolism; the citrate cycle; glycine, serine, and threonine metabolism; folate biosynthesis; alanine, aspartate, and glutamate metabolism; and one carbon pool by folate were remarkedly downregulated in the HFD group, and the first one was reversed by the SM10, LB8, ZF21, and LC49 treatments. Moreover, glutathione metabolism, BCAA degradation, tryptophan metabolism, and the citrate cycle were also restored in the LC49 group, and the first three were even higher than those in the NC group. The rest of the 17 differential pathways were upregulated in the HFD group; the changes in pentose and glucuronate interconversions and glycerolipid metabolism were reversed by the QL33, LB8, ZF21, and LC49 strains. Additionally, LC49 treatment downregulated the pathways involved into the pentose phosphate pathway; phenylalanine, tyrosine, and tryptophan biosynthesis; carbohydrate metabolism; and cysteine and methionine metabolism.

Next, alterations in SCFA production were determined to explore the impact of *F. prausnitzii* treatment on gut microbial metabolism. The HFD group showed remarkably lower levels of acetate, propionate, and butyrate than the NC group (Figure 5C–E; *p* < 0.01). However, the LC49 and LB8 strain treatments notably elevated the content of acetate, propionate, and butyrate compared with the HFD group. Furthermore, no statistically significant difference was observed in iso-butyrate levels among the NC, HFD, and *F. prausnitzii*-treated groups (Figure 5F; *p* > 0.05).

### 3.6. Correlation Analysis between the Phenotypes of NAFLD and Multiple Indicators in the Gut

**Figure 6:**
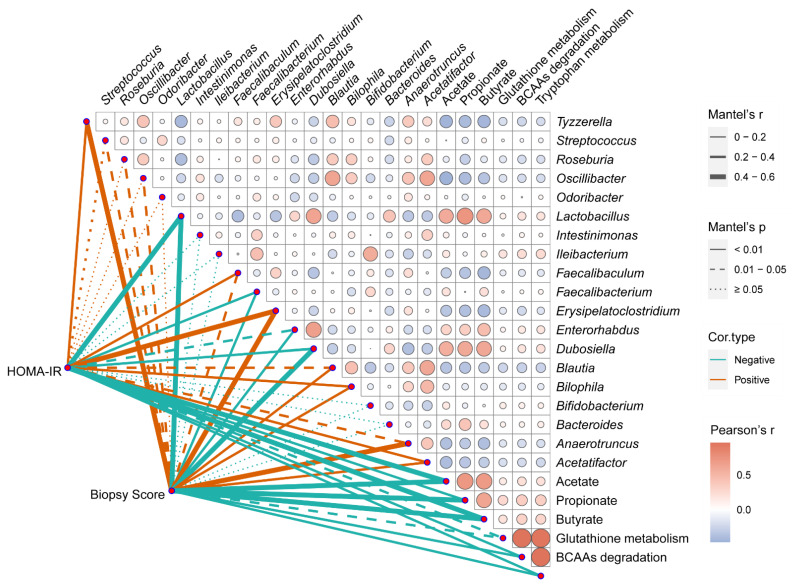
Correlation analysis of representative phenotypes of NAFLD to gut microbial indicators. The color and size of the circles in the matrix code indicate the correlation level. The thickness, color, and type (solid or dot) of the lines represent the correlation level, correlation type, and *p* value level, respectively. The correlation analyses in intra-matrix or inter-matrix are based on Pearson’s correlation coefficient and Mantel’s test, respectively. BCAAs, valine, leucine, and isoleucine degradation; HOMA-IR, homeostatic model assessment for insulin resistance; Biopsy score indicates the sum of the hepatic steatosis grade and the inflammation grade.

A correlation analysis was carried out to investigate the relation between the phenotypes of NAFLD (HOMA-IR and biopsy score), microbial biomarkers, and three highly predicted functional pathways in the LC49 group. As presented in Figure 6, the HOMA-IR and biopsy score were significantly positively related to the relative abundance of *Tyzzerella*, *Faecalibaculum*, *Erysipelatoclostridium*, *Blautia*, *Bilophila*, *Anaerotruncus*, and *Acetatifacto* and negatively correlated with the relative abundance of *Lactobacillus*, *Dubosiella*, *Faecalibacterium*, and *Enterorhabdus* as well as the fecal levels of acetate, propionate, and butyrate. Additionally, the HOMA-IR and biopsy scores were negatively associated with glutathione metabolism, BCAA degradation, and tryptophan metabolism.

## 4. Discussion

The human gastrointestinal tract harbors trillions of microbes, which is approximately 10 times the number of all cells of the human body [26]. Undoubtfully, the gut microbiota is crucial for the host’s energy metabolism, thus affecting metabolic syndrome [27]. Numerous studies reported an apparently decreased *F. prausnitzii* abundance in NAFLD patients compared with healthy individuals [28,29,30]. Since *F. prausnitzii* is a dominant commensal bacterium in the human gut, we proposed a hypothesis on a potential causative link between *F. prausnitzii* and NAFLD. Thus, we examined the hepatoprotective effects of 12 human-derived *F. prausnitzii* strains on HFD-induced NAFLD. Our results revealed that *F. prausnitzii* strain-dependently ameliorated NAFLD phenotypes.

Dyslipidemia raises the risk of NAFLD and is closely related to cardiovascular mortality, which is the primary cause of death in NAFLD cases [31]. Approximately 20–80% of NAFLD patients have dyslipidemia, which is manifested by increased serum TC, TG, and LDL-C, and decreased HDL-C [32]. Here, we found that treatment with *F. prausnitzii* strains reversed dyslipidemia symptoms in the NAFLD mice, but strain-specific differences were observed (Figure 1E–I). Furthermore, *F. prausnitzii* supplementation reduced adipose tissue inflammation and expansion (Figure 2A–C and Figure S2). The adipose tissue expansion is concomitant with excessive lipolysis and the subsequently increased FFAs levels, causing dyslipidemia [33]. Therefore, the restoration of dyslipidemia and adipose tissue dysfunction indicate the beneficial effect of specific *F. prausnitzii* strains (A2-165, LB8, ZF21, PL45, and LC49) on NAFLD.

Earlier, the traditional “two-hit” model was commonly accepted for illustrating the pathogenesis of NAFLD [34]. Steatosis, mainly caused by insulin resistance, indicates the “first hit”, sensitizing the liver to the steatohepatitis and fibrosis resulting from “second hits”, such as oxidative stress, adipokines, inflammatory cytokines, and mitochondrial dysfunction [35]. Along with the experimental and theoretical developments, a “multiple-hit” theory was recently proposed. According to the “multi-hit” theory, NAFLD is induced by a combination of insults, including adipose-tissue-secreted hormones, insulin resistance, dietary factors, gut flora, and genetic and epigenetic factors [36]. Of note, liver fat accumulation still seems to represent the “first hit”. Our results indicate that *F. prausnitzii* supplementation, including A2-165, LB8, ZF21, PL45, and LC49, notably alleviated liver injury and hepatic steatosis, inflammation, and oxidative stress (Figure 3 and Figure S3). Furthermore, we demonstrated that *F. prausnitzii* treatments, except the JZ10, JZ27, QL33, and HW29 strains, significantly ameliorated the glucose intolerance caused by long-term feeding with an HFD (Figure 2D–H). The PCA analysis based on all NAFLD phenotypes suggested the beneficial effects of *F. prausnitzii* on NAFLD depend on the specific strain, with LC49 being the best and HW29 being the least beneficial strain (Figure S1). 

The gut microbiota, as a major node for the gut–liver axis, provides a direct target for probiotics to exert beneficial health effects [37,38]. Here we used 16s rRNA-amplicon sequencing to perform a comprehensive analysis of the gut microbiota. We observed no statistically significant differences in α-diversity between the NC, HFD, and *F. prausnitzii*-treated groups, indicating that an HFD might affect the overall composition of intestinal flora (Figure 4A,B) but not the richness. The β-diversity analysis indicated an apparent structural separation between the HFD and NC groups, and only the LC49 group was close to the NC group (Figure 4C). Moreover, long-term intervention with an HFD notably impacted the gut microbial composition at the phylum level, resulting in a higher Firmicutes/Bacteroides ratio compared to mice fed an NCD (Figure 4D,E; *p* < 0.01). This alteration in the Firmicutes/Bacteroides ratio caused by HFD feeding was reversed by *F. prausnitzii* LC49 but was aggravated by the JZ27, LA8, ZF21, and HW29 strains. Based on the results of the LEfSe analysis, *Tyzzerella*, *Roseburia*, *Odoribacter*, *Erysipelatoclostridium*, and *Faecalibaculum* were found to be the core microbiome in the HFD groups (Figure 4F), which is largely in agreement with previous studies [39,40,41,42]. The HOMA-IR and biopsy scores were significantly positively associated with the relative abundance of *Tyzzerella* (r = 0.39, *p* < 0.01; r = 0.45, *p* < 0.01), *Erysipelatoclostridium* (r = 0.42, *p* <0.01; r = 0.40, *p* < 0.05), and *Faecalibaculum* (r = 0.34, *p* <0.01; r = 0.27, *p* < 0.05), indicating that these three HFD-dependent taxa might be crucial for the pathogenesis of NAFLD (Figure 6). *Faecalibaculum* ingestion might cause depression-like phenotypes [43], whereas an increased abundance of *Erysipelatoclostridium* is strongly related to metabolic disorders [44]. A previous cohort study addressed the negative effects of *Tyzzeralla* on CVD [40]. *F. prausnitzii* strains specifically normalized these HFD-dependent microbial biomarkers, with the LC49 and LB8 strains performing the best. In addition, LC49 and LB8 promoted the bloom of *Lactobacillus*, which was significantly negatively correlated with *Tyzzerella* and *Faecalibaculum* (Figure 4H; r < −0.3, *p* < 0.01). *Lactobacillus* is reported to be beneficial for the prevention of NAFLD [17,45]. Interestingly, the *Bifidobacterium* abundance in *F. prausnitzii*-treated groups was remarkedly increased compared with the NC and HFD groups (Figure 4G), which can be explained by the potential cross-feeding relationship between *F. prausnitzii* and *Bifidobacterium* [46]. Only LC49 treatment significantly enriched *Dubosiella* and *Intestinimonas* (Figure 4G, *p* < 0.01), which are reported to be potential therapeutics for metabolic syndrome and diabetes mellitus [47]. Unexpectedly, four *F. prausnitzii*-treated groups exhibited a comparable abundance of *Faecalibacterium* with the HFD group, suggesting that human-derived *F. prausnitzii* strains might adapt the mouse gut in a strain-specific manner (Figure 4G and Figure S4). The different colonization efficiencies of *F. prausnitzii* strains might be attributed to the strain-dependent competition with the host microbiota for nutrients and adhesion sites [48]. These results from the gut microbiota analysis demonstrated that *F. prausnitzii* LB8 and LC49 restored the HFD-induced taxa to normal status, along with the increased abundance of genera that is beneficial for NAFLD prevention.

To further investigate the effects of *F. prausnitzii* treatment on the functional capabilities of the microbial community, a PICRUSt analysis was performed. Differential pathways between the NC and HFD groups were mainly involved in carbohydrate, lipid, and amino acid metabolism, and most abnormal alterations in NAFLD mice were reversed by LC49 and LB8 treatments. Interestingly, pathways involving glutathione metabolism, BCAA degradation, and tryptophan metabolism were more highly upregulated in the LC49 group than in the NC group. Glutathione is an important cellular free radical scavenger, whereas BCAAs act as important mediators of metabolic health [49,50]. It has been demonstrated that microbiota-derived tryptophan metabolites, such as indole-3-acetate, tryptamine, and indole-3-carbinol, can reduce the pathology of NAFLD through the aryl hydrocarbon receptor (AHR) pathway [51,52]. The results of the functional pathways suggested that the strain-specific effect of *F. prausnitzii* on NAFLD might be due to certain metabolites derived from the gut microbiota. Auger et al. demonstrated that the different anti-inflammatory properties of *F. prausnitzii* are attributed to the intraspecific diversity of its metabolite, microbial anti-inflammatory molecule (MAM) [53]. Therefore, an alteration of gut microbial functional pathways might be associated with the beneficial effect of the LB8 and LC49 strains on NAFLD. 

Microbiota-derived SCFAs play crucial roles in maintaining hepatic energy homeostasis. SCFAs can affect appetite and calorie consumption by stimulating the secretion of peptide YY (PYY), glucagon-like peptide (GLP-1), and leptin [3]. Moreover, SCFAs may have a positive impact on NAFLD by affecting energy expenditure and lipid metabolism. An oral treatment with propionate or butyrate increases energy expenditure and lipid oxidation [54,55], which is potentially beneficial for fat accumulation in the liver. Moreover, acetate supplementation prevents adiposity by promoting adipose tissue browning [56]. We found that treatment with *F. prausnitzii* LC49 and LB8 notably increased the content of acetate, propionate, and butyrate compared with the HFD group (Figure 5C–F), indicating that gut microbiota-derived SCFAs were potential mediators for LB8 and LC49 strains exerting an anti-NAFLD effect.

Based on a comprehensive analysis of NAFLD phenotype hallmarks, SCFA metabolism, and gut microbial analysis, the capacity of *F. prausnitzii* strains to prevent HFD-induced NAFLD were found to be varied. Given the different capabilities of producing butyrate among these *F. prausnitzii* strains and the significant negative correlation between butyrate and two representative NAFLD indicators (HOMA-IR and biopsy score, Figure 6), we considered the butyrate-producing ability as the main reason for the strain-specific beneficial effect of *F. prausnitzii* on NAFLD. However, our previous work showed that the HW29 strain produced higher butyrate levels than ZF21 [57], but the protective effect on NAFLD was not comparable to that of ZF21 (Figure S1). In addition, LC49, the most effective strain, showed a significantly higher enrichment in pathways involving tryptophan metabolism, glutathione metabolism, and BBCA degradation than the HFD group (Figure 5B). Accordingly, the combined effect of SCFAs and other potential microbiota-derived metabolites might be responsible for the protective effect of *F. prausnitzii* strains on NAFLD, and the involvement of the gut microbiota is critical, as indicated by the results from the correlation analysis (Figure 6).

In summary, our results demonstrated that *F. prausnitzii* LB8 and LC49 significantly ameliorated the symptoms associated with a mouse model of NAFLD, restored the gut microbial dysbiosis, and modulated the gut microbial functional pathways and SCFA production. Nevertheless, the detailed mechanisms underlying the microbiota-derived SCFAs, tryptophan metabolites, or other substances as key mediators of the anti-NAFLD effect of the LB8 and LC49 strains require further investigation. Collectively, the findings of this study indicate the potential of the LB8 and LC49 strains as probiotic agents for NAFLD prevention.

## Supplementary Materials

The following supporting information can be downloaded at: https://www.mdpi.com/article/10.3390/nu14142945/s1, Figure S1: Score plots of PCA for all NAFLD phenotype measurements, Figure S2: Relative expression of TNF-α (A) and IL-6 (B) mRNA in adipose tissue, Figure S3: *F. prausnitzii* treatment prevented hepatic fibrosis in NAFLD mice, Figure S4: Quantification of *F. prausnitzii* in feces using RT-qPCR, Table S1: The information of *F. prausnitzii* strains used in the present study, Table S2: Sequence of primers used in gene expression studies. Supplementary References [58,59,60,61].
