# Supplementary material for: Specific Strains of Faecalibacterium prausnitzii Ameliorate Nonalcoholic Fatty Liver Disease in Mice in Association with Gut Microbiota Regulation
Source: Nutrients. 2022 Jul 19;14(14):2945. doi: 10.3390/nu14142945 (PMC9325077; doi:10.3390/nu14142945)
Supplement: Supplementary file 1 [file nutrients-14-02945-s001.zip › nutrients-1780152-supplementary.pdf]

Supplementary materials

Bacterial suspension preparation of F. prausnitzii strains

Prior to use in animal experiments, all strains were reactivated in an anaerobic workstation (Electrotek 400TG workstation, Eletrotek, West Yorkshire, UK) three consecutive times using 2% (v/v) inoculum in a modified M2GSC medium, containing (per 1000 mL) 5 g of yeast extract, 4 g of NaHCO3, 5 g of glucose, 2 g of fructose, 2 g of cellobiose, 10 g of casitone, 1.0 g of cysteine, 0.45 g of K2HPO4, 0.45 g of KH2PO4, 0.9 g of NaCl, 0.9 g of (NH4)2SO4, 0.09 of g MgSO4, 0.09 g of CaCl2, 1 mg of resazurin, and10 mL of clarified rumen fluid. Subsequently, the cultured bacterial cells were prepared at the anaerobic workstation using a filter upper cup (0.22 μm, Zhejiang Sorfa Life Science Research Co. Ltd, Zhejiang, China). Finally, the cell solutions used for oral administration were obtained by suspending the cultured bacterial cells in PBS (supplemented with 0.05% L-cysteine) at an approximate cell density of 4×10^9 CFU mL^{-1}.

Gut microbiota analysis

Total DNA was extracted from stool samples using a Fast DNA Stool Kit (MP Biomedicals, CA, USA). The V3-V4 region of 16s rDNA was amplified by primers 341F and 806R, and barcode is six-base unique to each sample. Amplicons were extracted form 2 % agarose gels and purified using the TIANgel Mini Purification Kit (TIANGEN, Beijing, China). Finally, the purified amplicons were pooled in equal concentration, and paired-end sequencing was performed using the Illumina MiSeq PE300 platform (Illumina, CA, USA).

Quantitative Insights into Microbial Ecology version 2 (QIIME2) platform was applied to process the raw data of sequencing [58]. Operational taxonomic units (OTUs) were identified using the criterion of 97 % nucleotide identity based on a reference dataset from the SILVA database v132.99. The differential abundance of each OTU was analyzed in QIIME2 using the statistical framework "Analysis of Composition Microbiomes" (ANCOM) at different taxonomic levels. α-Diversity was evaluated by evenness and species richness from the rarefied OTU and indicated as the Shannon index and observed OTUs. β-Diversity was estimated by Bray-Crutis distances and visualized by principal component analysis (PCA). Microbial biomarkers were differentiated using linear discriminant analysis (LDA) effect size (LEfSe) (Wilcoxon-rank sum test, $P < 0.05$, LDA score > 2.0) and visualized using a taxonomic cladogram tree (http://huttenhower.sph.harvard.edu/galaxy). The phylogenetic investigation of communities by reconstruction of unobserved states (PICRUSt) platform was used to predicted the functional composition of a metagenome from 16S rRNA sequences [59]. The network was constructed with Spearman's correlation coefficients and visualized using Gephi (v0.9.2).

***Real-time qPCR analysis* (*RT-qPCR*)**

Quantification of F. praunitzii in feces

A2-165 (reference strain of *F. prausnitzii*) broth culture was firstly diluted into 10^{-1}~10^{-4}. Colony-forming units (CFU) for each dilution sample were determined, followed by the DNA extraction using TIANamp Bacterial DNA kit (Tiangen Biotech, Beijing, China). Total DNA from feces was extracted using a Fast DNA Stool Kit (MP

Biomedicals, CA, USA). RT-qPCR was performed on a BioRad-CFX384 system using SYBR Green Supermix (Bio-Rad, USA) and specific prime for *F. prausnitzii* (FPR-2F: 5'-GGAGGAAGAAGGTCTTCGG-3'; Fprau-645R: 5'-AATTCCGCCTACCTCTGCACT-3') [60]. Based on the RT-qPCR analysis of A2-165 cultures, a calibration curve was constructed using the Log_{10}CFU and Cq value. Quantification of *F. praunitzii* in feces was calculated using the calibration curve.

Gene expression of TNFα and IL-6

The total RNA in the epidydimal adipose tissue was extracted using Trizol regent (Invitrogen, USA). The complementary (cDNA) was synthesized using the HiScript II Q Select RT SuperMix (Vazyme, China). RT-qPCR was performed on a BioRad-CFX384 system using SYBR Green Supermix (Bio-Rad, USA) under the following schedule: preincubation 95 °C for 5 min, 40 amplification cycles consisting of denaturation at 95 °C for 30 s, 58-60 °C for 20 s and 72 °C for 30s. The sequence of primers used in this study is listed in Table S2. β-actin was used as a housekeeping gene for quantification. The relative mRNA expression levels of the target genes were calculated using the $2^{-\Delta\Delta Ct}$ method.

Table S1. The information of *F. prausnitzii* strains used in the present study

Strain name	Abbreviation	Source (age, gender)	Isolated location
DSM 17677	A2-165	34, female	Scotland, UK
FJNJZ1Y10	JZ10	25, male	Wuxi, Jiangsu, China
FJNJZ1Y27	JZ27	25, male	Wuxi, Jiangsu, China

FJNLA1Y08	LA8	28, male	Wuxi, Jiangsu, China
FJNLB1Y08	LB8	28, male	Wuxi, Jiangsu, China
FJNQL1Y13	QL13	25, female	Wuxi, Jiangsu, China
FJNQL1Y33	QL33	25, female	Wuxi, Jiangsu, China
FJNSM1Y10	SM10	28, female	Wuxi, Jiangsu, China
FJNZF1Y21	ZF21	36, male	Wuxi, Jiangsu, China
FJNHW1Y29	HW29	31, male	Wuxi, Jiangsu, China
FWXPL1Y45	PL45	48, female	Wuxi, Jiangsu, China
FWXLC1Y49	LC49	45, male	Wuxi, Jiangsu, China

Table S2 Sequence of primers used in gene expression studies

Gene Name	Primer Sequence	Reference
TNF-α	F: 5’- TGATCCGCGACGTGGAA -3’<br>R: 5’- ACCGCCTGGAGTTCTGGAA -3’	[61]
IL-6	F: 5’-CCAGTTGCCTTCTTGGGACT-3’<br>R: 5’-GGTCTGTTGGGAGTGGTATCC-3’	[61]
β-actin	F: 5’-CTGCGTTTTACACCCTTTCTTTG-3’<br>R: 5’- GCCATGCCAATGTTGTCTCTTAT -3’	[61]

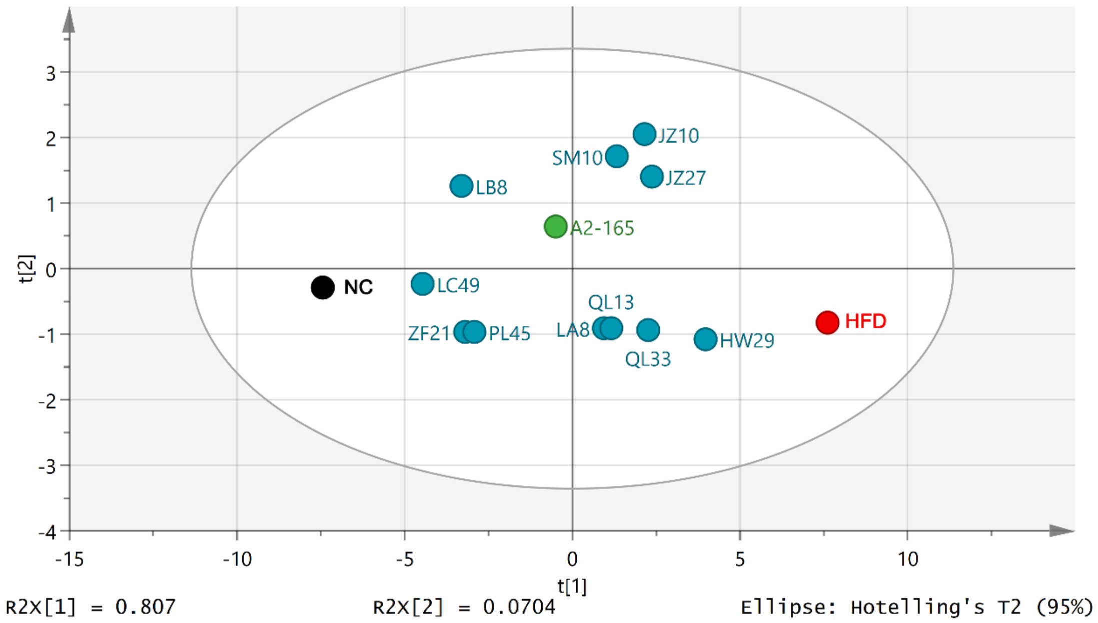

Figure S1. Score plots of PCA for all NAFLD phenotype measurements

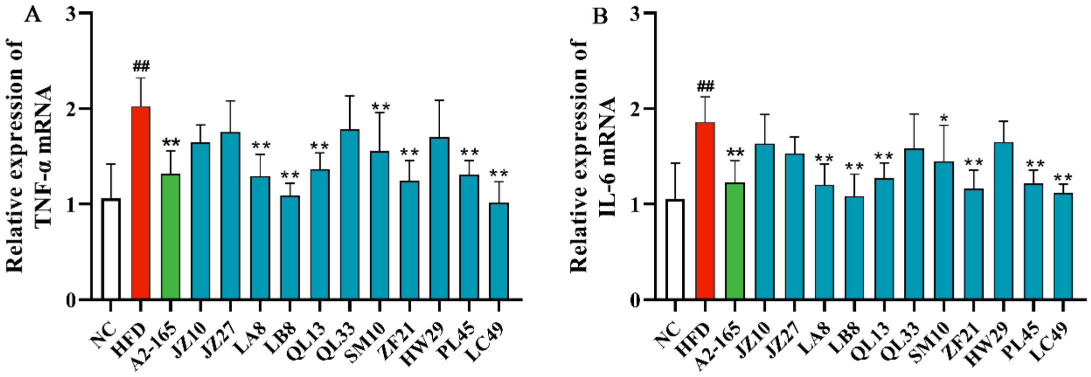

Figure S2. Relative expression of TNF-α (**A**) and IL-6 (**B**) mRNA in adipose tissue. Data are shown as means with SD; # $P < 0.05$, ## $P < 0.01$ vs. NC group, unpaired Student's t-test; *$P < 0.05$, **$P < 0.01$ vs. HFD group, one-way ANOVA followed by Dunnett's test.

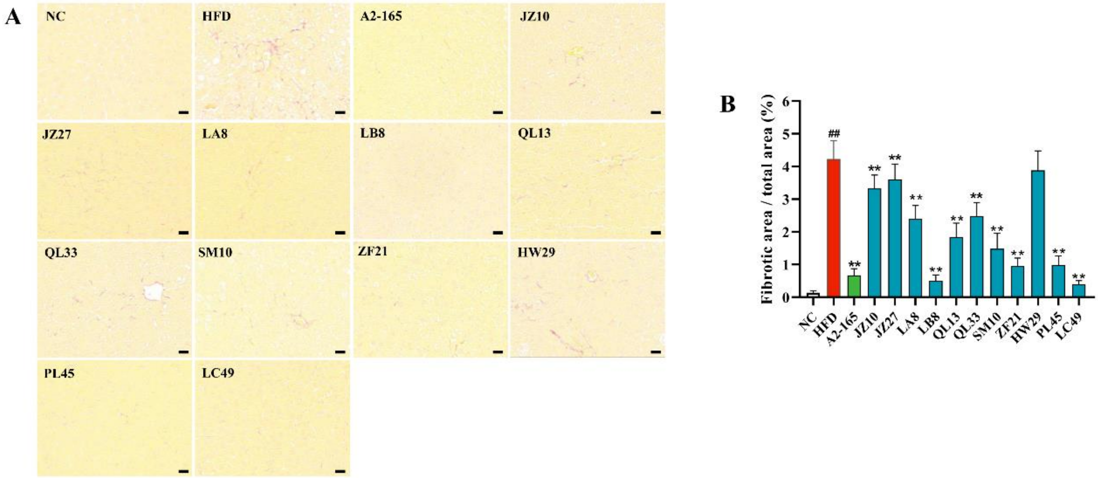

Figure S3. *F. prausnitzii* treatment prevented hepatic fibrosis in NAFLD mice. (**A**) Representative Sirius Red staining of liver (400 ×, scale bars represent 20 μm); (**B**) Quantification of fibrotic area in (**A**). Data are shown as means with SD; # $P < 0.05$, ## $P < 0.01$ vs. NC group, unpaired Student's t-test; *$P < 0.05$, **$P < 0.01$ vs. HFD group, one-way ANOVA followed by Dunnett's test.

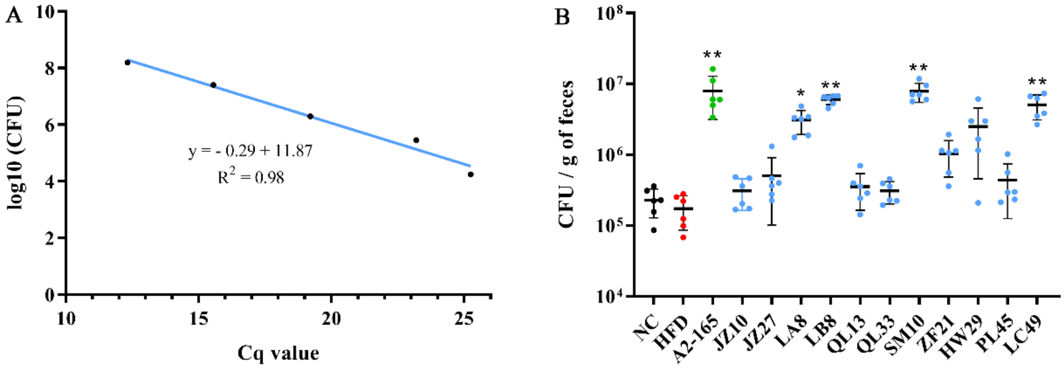

Figure S4. Quantification of *F. prausnitzii* in feces using RT-qPCR. (**A**) standard curve for A2-165. (**B**) Enumeration of *F. prausnitzii* in the feces of mice. Data are shown as means with SD; # $P < 0.05$, ## $P < 0.01$ vs. NC group, unpaired Student's t-test; *$P < 0.05$, **$P < 0.01$ vs. HFD group, one-way ANOVA followed by Dunnett's test.

References

58. Langille, M.G.I.; Zaneveld, J.; Caporaso, J.G.; McDonald, D.; Knights, D.; Reyes, J.A.; Clemente, J.C.; Burkepile, D.E.; Vega Thurber, R.L.; Knight, R.; et al. Predictive functional profiling of microbial communities using 16S rRNA marker gene sequences. *Nat. Biotechnol.* **2013**, 31, 814–821.
59. Bolyen, E.; Rideout, J.R.; Dillon, M.R.; Bokulich, N.A.; Abnet, C.C.; Al-Ghalith, G.A.; Alexander, H.; Alm, E.J.; Arumugam, M.; Asnicar, F.; et al. Reproducible, interactive, scalable and extensible microbiome data science using QIIME 2. *Nat. Biotechnol.* **2019**, 37, 852–857.

60. Fitzgerald, C.B.; Shkoporov, A.N.; Sutton, T.D.S.; Chaplin, A.V.; Velayudhan, V.; Ross, R.P.; Hill, C. Comparative analysis of Faecalibacterium prausnitzii genomes shows a high level of genome plasticity and warrants separation into new species-level taxa. *BMC Genomics* **2018**, 19, 1-20.
61. Xiong, X.-Q.; Geng, Z.; Zhou, B.; Zhang, F.; Han, Y.; Zhou, Y.-B.; Wang, J.-J.; Gao, X.-Y.; Chen, Q.; Li, Y.-H.; et al. FNDC5 attenuates adipose tissue inflammation and insulin resistance via AMPK-mediated macrophage polarization in obesity. *Metabolism* **2018**, 83, 31–41.
